# Professor John Michael Maisch, M. D.

**Published:** 1893-11

**Authors:** 


PROFESSOR JOHN MICHAEL MAISCH, M. D., who occupied the chair of the Materia Medica and Botany in the Philadelphia College of Pharmacy, died on Sunday, September 10, 1893, at his residence, No. 753 North 40th street.

Professor Maisch was a native of Hana-on-the-Main, Germany, where he was born in 1831. He came to this country in 1850, and has taught pharmacy for a generation both in New York and Philadelphia. He was also editor of the American Journal of Pharmacy, and revised the last edition of Griffith's Medical Formulary. He edited, jointly with Professor Stillé, four editions of the National Dispensatory, and also the Organic Materia Medica, of which a notice appears in this number of the JOURNAL. He was an acknowledged authority in the department in which he has spent so many years teaching and writing.

